# Low-dose intrathecal morphine versus intrathecal fentanyl for post-caesarean analgesia in a resource-constrained setting: a pragmatic randomised trial

**DOI:** 10.1186/s12871-026-04034-0

**Published:** 2026-06-23

**Authors:** Sean Chetty, Fathima Paruk, Peter Kamerman

**Affiliations:** 1 Department of Anaesthesiology & Critical Care, Faculty of Medicine and Health Sciences, Stellenbosch University, Cape Town, South Africa; 2 Department of Critical Care and Emergency Medicine, Faculty of Health Sciences, University of Pretoria, Pretoria, South Africa; 3 Department of Physiology, School of Biomedical Sciences, Faculty of Health Sciences, University of the Witwatersrand, Johannesburg, South Africa

**Keywords:** Caesarean Section, Spinal Anaesthesia, Morphine, Fentanyl, Postoperative Pain, Resource-constrained setting

## Abstract

**Background:**

Intrathecal morphine is recommended for post-caesarean analgesia, but its implementation remains inconsistent in resource-constrained obstetric services, where short-acting intrathecal fentanyl is often favoured instead because of familiarity and perceived simplicity of use. We compared two low doses of intrathecal morphine with intrathecal fentanyl in women undergoing caesarean delivery under spinal anaesthesia in a South African public-sector hospital.

**Methods:**

This was a single-centre, participant- and outcome-assessor-blinded, parallel-group pragmatic randomised trial. Women undergoing caesarean delivery under single-shot spinal anaesthesia were allocated to hyperbaric bupivacaine 0.5% 1.8 ml mixed with intrathecal morphine 100 µg (M100), intrathecal morphine 50 µg (M50), or intrathecal fentanyl 25 µg (F25). All participants received postoperative intravenous (IV) morphine patient-controlled analgesia (PCA) and rectal indomethacin. The primary outcome was cumulative 24-hour IV PCA morphine consumption. Secondary outcomes were morphine consumption for 0–12 h and 12–24 h, pain numerical rating scale (NRS) scores at rest and with cough at 12 and 24 h, perceived pain relief over 24 h, and whether participants wanted more analgesic treatment. The prespecified primary analysis was per protocol, with a supportive intention-to-treat analysis.

**Results:**

One hundred women were randomised. Ninety-three comprised the per-protocol efficacy cohort (M100 *n* = 32, M50 *n* = 29, F25 *n* = 32). Median 24-hour IV PCA morphine consumption differed significantly between groups: 12.5 mg (Q1, Q3: 6.0, 20.25) in M100, 15.0 mg (9.0, 25.0) in M50, and 26.0 mg (16.5, 38.5) in F25 (*p* < 0.001). After Holm adjustment for the three pairwise comparisons at the primary endpoint, the morphine groups required less rescue opioid than F25 (M100 vs. F25 adjusted *p* = 0.00081; M50 vs. F25 adjusted *p* = 0.019), whereas M100 and M50 did not differ (adjusted *p* = 0.326). Hodges-Lehmann estimates of the median difference in 24-hour morphine consumption were − 13.0 mg (95% CI -20.0 to -7.0) for M100 vs. F25 and − 10.5 mg (95% CI -18.0 to -3.0) for M50 vs. F25, and − 3.0 mg (95% CI -8.0 to 3.0) for M100 vs. M50. The same directional pattern was present for 0–12 h and 12–24 h. Pain scores at rest and with cough did not differ significantly between groups at either postoperative assessment. Perceived pain relief over 24 h was similar across groups, and the proportion of women who wanted more analgesic treatment did not differ significantly. The intention-to-treat analysis was concordant with the per-protocol analysis.

**Conclusions:**

In this single-centre pragmatic trial conducted in a South African public-sector hospital, low-dose intrathecal morphine at both 50 µg and 100 µg reduced 24-hour postoperative opioid requirements after caesarean delivery compared with intrathecal fentanyl 25 µg. These findings additionally support 50 µg intrathecal morphine as an effective and implementable option for post-caesarean analgesia in resource-constrained obstetric settings.

**Trial registration:**

ClinicalTrials.gov NCT02577809 (retrospectively registered 10 October 2015).

**Supplementary Information:**

The online version contains supplementary material available at https://doi.org/10.1186/s12871-026-04034-0.

## Background

Caesarean delivery is a high-volume operation in which the quality of postoperative analgesia matters well beyond pain intensity alone [1]. Poorly controlled post-caesarean pain impairs mobilisation, breastfeeding, maternal-infant interaction, sleep, and overall recovery [2]. Current procedure-specific guidance therefore places neuraxial morphine at the centre of multimodal post-caesarean analgesia. The updated PROSPECT guideline recommends intrathecal morphine 50–100 µg together with paracetamol, non-steroidal anti-inflammatory drugs, and intravenous dexamethasone, whilst the SOAP consensus statement supports low-dose neuraxial morphine with risk-stratified monitoring in appropriate obstetric patients [3, 4]. Nevertheless, concern about delayed respiratory depression remains an important barrier to neuraxial morphine implementation, even though clinically significant respiratory depression appears uncommon after low-dose neuraxial morphine in appropriately selected obstetric patients when risk-stratified monitoring is used [5]. 

The existence of guidelines does not guarantee implementation. In the international PAIN OUT caesarean registry, only a minority of women received all recommended perioperative pain-care elements, and further greater adherence to evidence-based care was associated with better pain-related outcomes after caesarean section. In African and other low-resource settings, this evidence-practice gap is especially relevant because analgesic choices are shaped not only by efficacy, but also by staffing, monitoring capacity, drug availability, knowledge levels and local preferences [6, 7]. 

Intrathecal opioid choice remains central to this evidence-practice gap. Intrathecal fentanyl is a familiar drug, it is known to improve intraoperative conditions, and can reduce the need for supplemental intraoperative analgesia, but its postoperative analgesic duration is limited [8]. By contrast, low-dose intrathecal morphine provides more durable analgesia and remains the reference neuraxial opioid for caesarean delivery [3, 9]. Dose-response work and meta-analyses suggest that the clinically useful range of intrathecal morphine lies within the low-dose window, and that escalation above this range offers diminishing efficacy gains while increasing adverse effects, such as delayed respiratory depression, pruritus and nausea. More recently, the 2025 Hatter trial found 50 µg intrathecal morphine non-inferior to 100 µg within a contemporary multimodal regimen, reinforcing the notion that the dose question also remains clinically relevant [8, 10–13]. 

The choice of intrathecal opioid strategy is particularly important in African public-sector obstetric services. Evidence for post-caesarean analgesia remains dominated by higher-resource environments, whereas implementation in African public hospitals must account for ward delays, limited monitoring capacity, and the need for simple, low-cost, reliable interventions. This is not merely theoretical. At Tygerberg Hospital, a 2025 quality-improvement analysis showed that rebound pain after spinal caesarean section remained common despite updated local guidelines, and that partial rather than full implementation of multimodal analgesic strategies appeared to explain ongoing pain burden [6, 7, 14]. 

The present study was designed to address that practice implementation gap. Rather than asking only whether one intrathecal morphine dose is pharmacologically superior to another, it asked whether low-dose intrathecal morphine offers a pragmatic postoperative advantage over intrathecal fentanyl in a South African public-sector hospital. The primary objective was to compare the cumulative 24-hour intravenous (IV) patient controlled analgesia (PCA) morphine consumption after intrathecal morphine 100 µg, intrathecal morphine 50 µg, and intrathecal fentanyl 25 µg in women undergoing caesarean delivery under spinal anaesthesia. Secondary objectives comprised the comparison of opioid consumption during the first and second 12-hour postoperative epochs, pain scores at rest and with cough, patient-reported percentage pain relief, and whether patients wanted more pain treatment. We hypothesised that both low-dose intrathecal morphine regimens would reduce postoperative opioid requirements compared with intrathecal fentanyl, and that 50 µg would provide efficacy comparable to 100 µg. Although conducted at a single South African public-sector hospital, the study was designed to address a broader problem of how to implement guideline concordant post-caesarean analgesia in resource-constrained obstetric services.

## Methods

### Study design and reporting

This was a single-centre, participant- and outcome-assessor-blinded, parallel-group randomised trial conducted at Rahima Moosa Mother and Child Hospital (RMMCH), Johannesburg, South Africa, between July and September 2015. The report was prepared in accordance with CONSORT principles for randomised trials. There was no patient or public involvement in the design, conduct or reporting of this study.

### Ethics approval and trial registration

The study was approved by the Human Research Ethics Committee of the University of the Witwatersrand (M141181) and conducted in accordance with the Declaration of Helsinki on Ethical Principles for Medical Research involving human subjects and Good Clinical Practice. Written informed consent was obtained from participants before enrolment. The trial was retrospectively registered at ClinicalTrials.gov (NCT02577809) on 10 October 2015.

### Setting

RMMCH is a tertiary public-sector maternity hospital in Johannesburg, South Africa, with approximately 12,000 deliveries per year and a caesarean delivery rate of about 30% at the time of the study.

### Participants

Women aged 18 years or older undergoing elective or emergency caesarean delivery under single-shot spinal anaesthesia were eligible. Consecutive eligible women were approached preoperatively. Opioid-naïve status was not independently verified as a separate eligibility criterion.

Exclusion criteria were:

- refusal or inability to provide informed consent.
- severe pre-eclampsia or eclampsia.
- inability to understand PCA use despite instruction.
- conversion to general anaesthesia.
- supplemental intraoperative intravenous opioid administration.
- major intraoperative obstetric complications.
- postoperative maternal ICU or high-care admission.
- postoperative circumstances preventing valid maternal follow-up, including prolonged neonatal separation.

### Randomisation and allocation concealment

Participants were randomised by the principal investigator in a 1:1:1 ratio using a computer-generated blocked randomisation sequence generated from www.sealedenvelope.com [15].Allocation was concealed in sealed opaque envelopes opened only after enrolment.

### Blinding

Participants and postoperative outcome assessors were blinded to treatment allocation. After enrolment, the principal investigator handed the sealed allocation envelope to the attending anaesthetist providing clinical care. The attending anaesthetist prepared and administered the intrathecal solution and was aware of allocation because group assignment determined drug preparation. None of the authors administered the intrathecal study drug. The principal investigator and research assistants who collected postoperative outcome data remained blinded to treatment allocation. The trial is therefore most accurately described as participant- and outcome-assessor-blinded, rather than double-blind.

### Interventions

All participants received spinal anaesthesia with 1.8 ml hyperbaric bupivacaine 0.5%, combined with one of three intrathecal opioid regimens:

- M100: morphine 100 µg.
- M50: morphine 50 µg.
- F25: fentanyl 25 µg.

The total intrathecal volume was standardised to 2.3 ml for all groups. Preservative-free morphine was used for intrathecal morphine administration.

### Perioperative and postoperative analgesic management

All patients received standard intraoperative monitoring. Hypotension was treated according to local practice.

Postoperatively, all women received IV morphine PCA and rectal indomethacin according to local practice. The PCA solution contained morphine 1 mg.ml^− 1^ and was programmed to deliver a 1 mg bolus, 5-minute lockout, maximum 10 mg.h^− 1^, with no background infusion. All participants were taught how to use the PCA pump before surgery and were instructed again in the recovery area before PCA use continued on the ward. Correct use was confirmed pragmatically by ensuring that the participant understood that she should press the PCA demand button when analgesia was required. PCA use was otherwise monitored through the pump-recorded morphine consumption at the 12- and 24-hour assessments. No separate formal PCA adherence score was collected.

After surgery, participants were transferred to the recovery area for routine postoperative observation. Once standard recovery-area discharge criteria were met, they were transferred to the obstetric ward, where routine ward observations continued according to local practice. Study-specific postoperative assessments were performed at 12 ± 1 h and 24 ± 1 h after surgery and included PCA morphine consumption, pain scores, sedation score, respiratory rate, nausea/vomiting, and pruritus.

### Outcomes

The primary outcome was cumulative IV PCA morphine consumption for each of the 3 groups during the first 24 h after surgery.

Secondary outcomes were:

- IV PCA morphine consumption during the first 12 postoperative hours.
- IV PCA morphine consumption during 12–24 h.
- pain intensity at rest and with cough at 12 and 24 h, measured on an 11-point numerical rating scale (0 = no pain, 10 = worst pain imaginable).
- patient-reported percentage pain relief over the first 24 h.
- whether the participant wanted more analgesic treatment.

Safety observations, including sedation, respiratory rate, nausea/vomiting, and pruritus, were collected prospectively at 12 ± 1 h and 24 ± 1 h after surgery. Because the study was powered for analgesic efficacy rather than for uncommon adverse events, safety outcomes are reported descriptively and are not used for definitive comparative safety inference.

### Data collection

Baseline data included age, weight, parity, previous pregnancy losses, ASA physical status, baseline blood pressure, urgency of surgery, and perioperative course. Postoperative follow-up was performed at 12 ± 1 h and 24 ± 1 h using structured trial data forms and the PAIN OUT questionnaire framework [16]. Recorded postoperative variables included PCA morphine use, pain scores, perceived pain relief, and whether the participant wanted more pain treatment.

### Sample size

Sample size was calculated for the primary outcome of postoperative opioid requirement using an F-test with repeated measures across two postoperative time periods and three treatment groups, assuming correlation of 0.5, a small effect size (Cohen’s f = 0.2), and 90% power. This yielded 28 participants per group. To allow for attrition and exclusions, the target sample size was increased to 100 participants.

### Statistical analysis

The prespecified primary efficacy analysis was performed in the per-protocol cohort, defined as randomised participants who received study treatment and had evaluable maternal postoperative follow-up according to protocol. This approach was chosen a priori because the primary endpoint depended on valid maternal postoperative assessment, and six post-randomisation exclusions were prespecified because prolonged neonatal higher-level care precluded such assessment; one additional participant withdrew before the first postoperative assessment. These exclusions were determined before unblinding and were not plausibly related to allocated treatment. A supportive intention-to-treat analysis including all randomised participants was also undertaken to assess the robustness of the findings.

Continuous non-parametric outcomes are presented as median (Q1, Q3) and categorical variables as number (percentage). Between-group comparisons for postoperative morphine consumption and pain scores were performed using the Kruskal-Wallis test. When the overall test was significant, pairwise comparisons were undertaken using the Wilcoxon rank-sum test. For the primary 24-hour endpoint, pairwise comparisons were interpreted only after a significant omnibus Kruskal-Wallis test, and Holm adjustment was applied across the three pairwise comparisons to control the family-wise error rate. The magnitude of the pairwise differences at the primary 24-hour endpoint was quantified using Hodges-Lehmann estimates of the median difference with 95% confidence intervals. Pairwise comparisons for the 0–12 h and 12–24 h epochs were treated as secondary supportive analyses and were not adjusted for multiplicity.

Categorical variables were compared using the Chi-squared test. For the intention-to-treat sensitivity analysis, missing 24-hour values were handled using last observation carried forward; for the single participant who withdrew before the 12-hour assessment, imputation used the group median at each time point from the group to which she had been randomised. All analyses were two-sided, and *p* < 0.05 was considered statistically significant.

## Results

### Participant flow and analysis populations

**Fig. 1:**
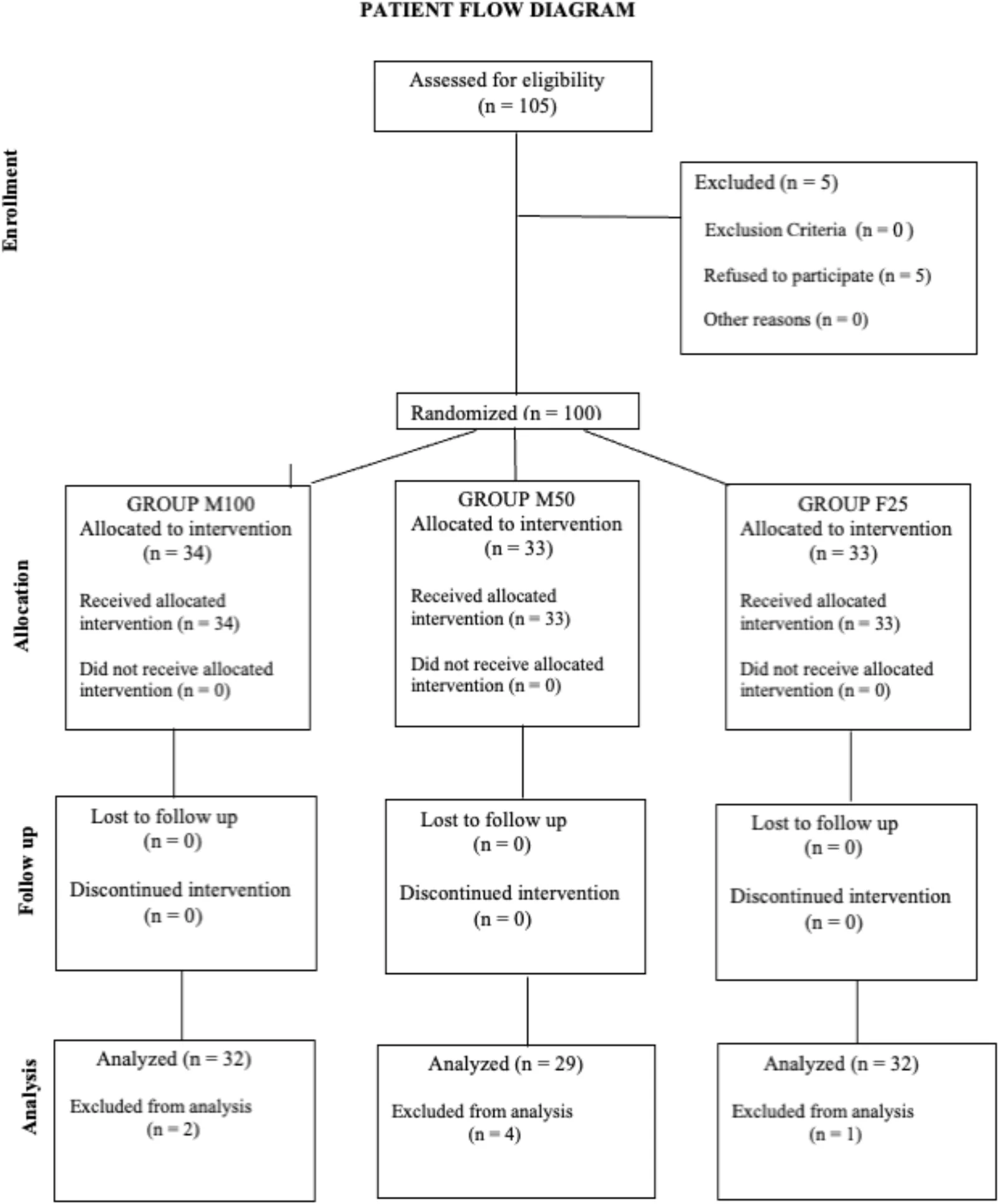
CONSORT flow diagram

Participant flow is shown in Fig. 1. A total of 100 women were randomised. Seven women were excluded from the per-protocol efficacy analysis: two in M100, four in M50, and one in F25. Six exclusions were due to prolonged neonatal higher-level care, which precluded valid maternal postoperative assessment according to the protocol; these comprised two participants in M100, three in M50, and one in F25. One additional participant in M50 withdrew after surgery before the first postoperative assessment. These post-randomisation exclusions were prespecified in the protocol and reflected inability to obtain valid maternal postoperative outcome data rather than treatment failure, crossover, or treatment-related withdrawal. The final per-protocol efficacy cohort therefore comprised 93 women: 32 in M100, 29 in M50, and 32 in F25.

### Baseline characteristics

The three groups were comparable at baseline. There were no statistically significant between-group differences in age, weight, primiparity, previous pregnancy loss, number of emergency procedures, or duration of surgery (Table 1).

**Table 1:** Baseline characteristics by randomised group

Intervention	Morphine 100 µg	Morphine 50 µg	Fentanyl 25 µg	
Number of Participants	32	29	32	
Age, mean (range), years	31 (21–40)	30 (23–39)	30 (21–41)	*P* = 0.88
Primiparity, n (%)	2 (6.3)	1 (3.4)	5 (15.6)	*P* = 0.20
Previous Pregnancy Loss, n (%)	6 (18.8)	4 (13.8)	6 (18.8)	*P* = 0.74
Weight, mean (range), kg	80.3 (49–101)	80.6 (50–106)	84.8 (55–164)	*P* = 0.89
Emergency Procedure, n (%)	9 (28.1)	10 (34.5)	11 (34.4)	*P* = 0.83
Surgery duration, median (range), min	55 (22–106)	65 (27–108)	55 (28–115)	*P* = 0.43

Values are mean (range), median (range), or n (%), as appropriate. P-values were derived using chi-squared tests for categorical variables and Kruskal-Wallis tests for continuous variables

### Primary outcome: cumulative 24-hour IV PCA morphine consumption

**Fig. 2:**
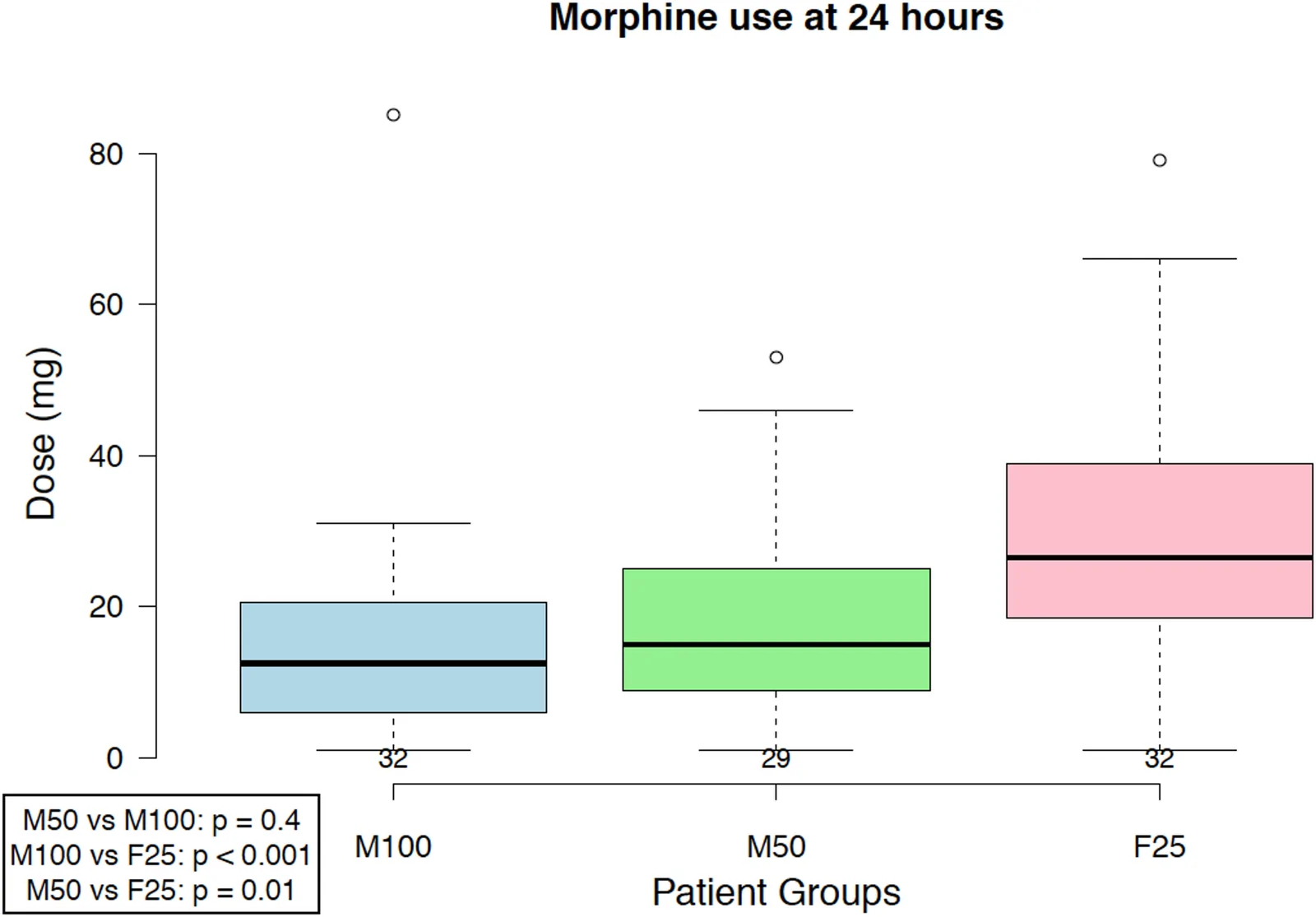
Box plot of cumulative 24-hour IV PCA morphine consumption by randomised group

Cumulative IV PCA morphine consumption over the first 24 postoperative hours differed significantly between groups (Table 2; Fig. 2). Median 24-hour morphine consumption was 12.5 mg (Q1, Q3: 6.0, 20.25) in M100, 15.0 mg (Q1, Q3: 9.0, 25.0) in M50, and 26.0 mg (Q1, Q3: 16.5, 38.5) in F25. The overall between-group comparison was significant (Kruskal-Wallis, *p* < 0.001).

**Table 2:** Postoperative IV PCA morphine consumption by randomised group

Outcome	M100 (*n* = 32)	M50 (*n* = 29)	F25 (*n* = 32)	Overall *p*-value
Morphine consumption, 0–12 h	8.0 (2.75, 12.0)	8.0 (4.0, 17.0)	16.0 (11.0, 25.5)	0.0021
Morphine consumption, 12–24 h	3.5 (1.0, 7.0)	5.0 (3.0, 7.0)	10.0 (4.75, 14.0)	0.0104
Morphine consumption, 0–24 h	12.5 (6.0, 20.25)	15.0 (9.0, 25.0)	26.0 (16.5, 38.5)	0.00078

Values are median (Q1, Q3), mg. Overall p-values were derived from the Kruskal-Wallis test. Pairwise comparisons for the primary 24-hour endpoint were performed using the Wilcoxon rank-sum test and adjusted using the Holm method across the three pairwise comparisons: M100 vs. F25, adjusted *p* = 0.00081; M50 vs. F25, adjusted *p* = 0.019; M100 vs. M50, adjusted *p* = 0.326. Pairwise comparisons for the 0–12 h and 12–24 h epochs were secondary supportive analyses and were not adjusted for multiplicity

The 0–12 h, 12–24 h, and 0–24 h values are medians calculated separately from participant-level distributions, therefore, the median 0–24 h value is not expected to equal the sum of the two epoch-specific medians

Pairwise comparisons showed that both intrathecal morphine groups required less rescue opioid than the fentanyl group, whereas there was no significant difference between the two morphine doses. After Holm adjustment for the three pairwise comparisons at the primary endpoint, the adjusted p-values were 0.00081 for M100 vs. F25, 0.019 for M50 vs. F25, and 0.326 for M100 vs. M50. Hodges-Lehmann estimates of the median difference in 24-hour morphine consumption were − 13.0 mg (95% CI − 20.0 to − 7.0) for M100 vs. F25, − 10.5 mg (95% CI − 18.0 to − 3.0) for M50 vs. F25, and − 3.0 mg (95% CI − 8.0 to 3.0) for M100 vs. M50; negative values indicate lower morphine consumption in the first-named group. These findings indicate that both low-dose intrathecal morphine regimens produced a clinically and statistically significant opioid-sparing effect compared with intrathecal fentanyl, with no evidence of additional efficacy from increasing the intrathecal morphine dose from 50 µg to 100 µg.

### Secondary efficacy outcomes: morphine consumption by postoperative epoch

The same efficacy pattern was present in both postoperative epochs (Table 2; Supplementary Figures S1 and S2). During the first 12 h after surgery, median IV PCA morphine consumption was 8.0 mg (Q1, Q3: 2.75, 12.0) in M100, 8.0 mg (Q1, Q3: 4.0, 17.0) in M50, and 16.0 mg (Q1, Q3: 11.0, 25.5) in F25. During the second 12 h, corresponding values were 3.5 mg (Q1, Q3: 1.0, 7.0), 5.0 mg (Q1, Q3: 3.0, 7.0), and 10.0 mg (Q1, Q3: 4.75, 14.0). Overall between-group comparisons were significant for both time periods. Pairwise Wilcoxon analyses again showed that M100 vs. F25 and M50 vs. F25 were significantly different at both epochs, whereas M100 vs. M50 was not. For the 0–12 h epoch, the pairwise p-values were 0.00055 (M100 vs. F25), 0.0192 (M50 vs. F25), and 0.406 (M100 vs. M50). For the 12–24 h epoch, the corresponding p-values were 0.00815, 0.0141, and 0.472. Thus, the opioid-sparing advantage of intrathecal morphine was established early and persisted throughout the 24-hour observation period.

### Pain scores

Pain outcomes are presented in Table 3. Pain scores at rest and with cough did not differ significantly between groups at either postoperative assessment. At 12 h, median pain at rest was 1.0 (Q1, Q3: 0.0, 2.25) in M100, 1.0 (0.0, 2.0) in M50, and 1.0 (1.0, 4.25) in F25 (Kruskal-Wallis test, *p* = 0.130), while median pain with cough was 2.0 (0.0, 5.0), 2.0 (1.0, 4.0), and 3.5 (1.0, 8.0), respectively (Kruskal-Wallis test, *p* = 0.088). At 24 h, median pain at rest was 2.0 (0.75, 3.25) in M100, 1.0 (1.0, 4.0) in M50, and 2.5 (1.0, 5.0) in F25 (Kruskal-Wallis test, *p* = 0.311), while median pain with cough was 3.0 (1.75, 6.0), 3.0 (2.0, 6.0), and 5.0 (2.0, 7.0), respectively (Kruskal-Wallis test, *p* = 0.082).

**Table 3:** Pain scores, perceived pain relief, and desire for additional pain treatment by randomised group

Outcome	M100 (*n* = 32)	M50 (*n* = 29)	F25 (*n* = 32)	Overall *p*-value
Pain at rest, 12 h (NRS 0–10)	1.0 (0.0, 2.25)	1.0 (0.0, 2.0)	1.0 (1.0, 4.25)	0.130
Pain with cough, 12 h (NRS 0–10)	2.0 (0.0, 5.0)	2.0 (1.0, 4.0)	3.5 (1.0, 8.0)	0.088
Pain at rest, 24 h (NRS 0–10)	2.0 (0.75, 3.25)	1.0 (1.0, 4.0)	2.5 (1.0, 5.0)	0.311
Pain with cough, 24 h (NRS 0–10)	3.0 (1.75, 6.0)	3.0 (2.0, 6.0)	5.0 (2.0, 7.0)	0.082
Perceived pain relief over 24 h, %	75 (40, 90)	70 (50, 90)	70 (40, 80)	0.77
Wanted more pain treatment, n/N (%)	13/32 (40.6%)	6/29 (20.7%)	15/32 (46.9%)	0.089

Values are median (Q1, Q3) unless otherwise stated. Desire for additional pain treatment is presented as n/N (%). Overall p-values for continuous outcomes were derived from the Kruskal-Wallis test. The *p*-value for the binary outcome was derived from the chi-squared test

### Patient-reported pain relief and desire for additional analgesic treatment

Perceived pain relief over 24 h was similar across groups, with median scores of 75.0% (Q1, Q3: 40.0, 90.0) in M100, 70.0% (50.0, 90.0) in M50, and 70.0% (40.0, 80.0) in F25 (Kruskal-Wallis, *p* = 0.770). The proportion of women who wanted more analgesic treatment was 40.6% in M100, 20.7% in M50, and 46.9% in F25 (Chi-squared test, *p* = 0.089).

### Descriptive safety observations

Descriptive safety observations are presented in Supplementary Table S1. Respiratory depression, defined as respiratory rate < 8 breaths/min, was not observed in any participant. Any nausea or vomiting occurred in 9/32 (28.1%) participants in M100, 9/29 (31.0%) in M50, and 9/32 (28.1%) in F25. Severe nausea or vomiting occurred in 5/32 (15.6%), 0/29 (0.0%), and 5/32 (15.6%), respectively. Any pruritus occurred in 12/32 (37.5%) participants in M100, 14/29 (48.3%) in M50, and 9/32 (28.1%) in F25, while severe pruritus occurred in one participant in each group. Any sedation was recorded in 7/32 (21.9%), 7/29 (24.1%), and 10/32 (31.2%) participants, respectively. Clinically significant sedation was uncommon. These observations should be interpreted descriptively because the trial was not powered for uncommon adverse events.

### Supportive intention-to-treat analysis

A supportive intention-to-treat analysis including all randomised participants yielded results consistent with the per-protocol analysis. Median 24-hour IV PCA morphine consumption was 13.5 mg (Q1, Q3: 6.25, 20.75) in M100, 15.0 mg (Q1, Q3: 9.0, 21.0) in M50, and 26.0 mg (Q1, Q3: 17.0, 40.0) in F25, with a significant overall between-group difference (Kruskal-Wallis test, *p* < 0.001).

Corresponding median IV PCA morphine consumption during 0–12 h was 9.0 mg (Q1, Q3: 3.0, 12.0) in M100, 8.0 mg (Q1, Q3: 5.0, 16.0) in M50, and 16.0 mg (Q1, Q3: 11.0, 27.0) in F25; during 12–24 h it was 4.5 mg (Q1, Q3: 1.25, 7.0), 6.0 mg (Q1, Q3: 3.0, 8.0), and 10.0 mg (Q1, Q3: 5.0, 14.0), respectively.

## Discussion

This pragmatic randomised trial demonstrated that, in women undergoing caesarean delivery in a South African public-sector hospital, intrathecal morphine at either 50–100 µg significantly reduced 24-hour postoperative IV PCA morphine requirements compared with intrathecal fentanyl 25 µg. The signal was not confined to the overall Kruskal-Wallis test: the pairwise comparisons demonstrated consistent separation of both morphine groups from fentanyl across the first 12 h, the second 12 h, and the full 24-hour period, while 50 µg and 100 µg did not separate from one another on efficacy grounds. This is the key clinical finding, because it identifies the benefit as a class effect of low-dose intrathecal morphine over a short-acting comparator, not as a dose-escalation effect within the morphine range.

This distinction matters when these data are placed beside the existing literature. Palmer’s dose-response study showed that analgesic benefit plateaued within the lower intrathecal morphine dose range, and Sultan’s meta-analysis later concluded that whilst higher doses may prolong analgesia, they achieve this at the cost of more pruritus and vomiting rather than a clear reduction in 24-hour opioid consumption [10, 11]. Carvalho and Tenório likewise reported comparable analgesic quality with 50 µg and 100 µg, and Hatter’s 2025 randomised trial found 50 µg non-inferior to 100 µg within a contemporary multimodal pathway [12, 13]. Our results are concordant with this low-dose signal, but they answer a more practice-facing question. Most earlier studies compared variable morphine dose strategies with one another in settings already committed to neuraxial morphine. By contrast, this trial asked whether a change in practice from intrathecal fentanyl to low-dose intrathecal morphine produces a pragmatic postoperative benefit in routine care. For that question, the answer is clearly affirmative.

The similarity in pain scores despite large between-group differences in opioid consumption is not contradictory. All women in this study had access to IV PCA morphine and were instructed in its use. Under those circumstances, pain scores reflect pain after self-titration, whereas cumulative opioid use reflects how much additional treatment was required to achieve that level of comfort. In a PCA-based framework, between-group pain-score differences are also likely to be compressed because participants titrate toward an acceptable comfort threshold. The clinical relevance of the opioid-sparing effect is therefore that women in the intrathecal morphine groups achieved similar pain scores with substantially less supplemental systemic opioid. This may reduce opioid burden, simplify rescue analgesic requirements, and support opioid stewardship, although this trial was not powered to demonstrate downstream reductions in uncommon adverse effects. This interpretation is reinforced by the similar perceived pain relief across groups.

The relatively frequent desire for additional analgesic treatment should therefore be interpreted cautiously. This binary patient-reported outcome does not necessarily indicate uncontrolled pain. It may also reflect expectations, timing of assessment, perceived control, communication, and the broader care experience. The finding also needs to be interpreted in the context of a postoperative regimen that did not include routine paracetamol or intravenous dexamethasone, both of which are now recommended as part of contemporary multimodal post-caesarean analgesia. Although the proportion of women wanting more pain treatment was numerically lowest in the M50 group, the overall comparison was not statistically significant and should not be used to infer a true between-group difference. More broadly, patient-reported pain outcomes after surgery are multifactorial and are shaped by expectations, perceived control, communication, and the wider care experience as well as by pain intensity itself [17]. 

The LMIC African and public-sector context is a major strength of this work. Contemporary guidance still recommends intrathecal morphine 50–100 µg, yet implementation remains incomplete even in current practice. The international PAIN OUT caesarean analysis showed that fuller adherence to evidence-based perioperative pain-care elements was associated with better outcomes, but only about one-fifth of women received complete recommended care [7]. More recent implementation work likewise showed that introducing intrathecal morphine into routine clinical practice still reduced opioid consumption and improved recovery [18]. In African hospitals, where practice is shaped by resource constraints as well as a paucity of local evidence, the more relevant question is not whether intrathecal morphine can work under ideal conditions, but whether a low-dose intrathecal morphine strategy is effective, feasible, and worth adopting in routine service platforms [6]. This randomised trial provides evidence that it is.

That point also explains why data collected a decade ago remain relevant. The relevance of this dataset does not depend on novelty in the narrow pharmacological sense, or on the postoperative regimen representing a fully optimised contemporary enhanced-recovery pathway. Its value lies in showing that a guideline-concordant, low-dose intrathecal morphine strategy produced meaningful opioid sparing in a South African public-sector maternity hospital. The gap between evidence and routine practice has not disappeared. Indeed, a recent South African rebound-pain study showed that, even after updated departmental guidelines, severe post-spinal caesarean pain remained common and partial implementation appeared to limit the full effect of the protocol [14]. Taken together, those findings and this trial make a coherent regional case that low-dose intrathecal morphine is effective, but implementation quality determines whether that efficacy is translated into routine patient benefit.

The practical implication is therefore stronger than a simple dose-comparison message. For low-resource obstetric services still relying on intrathecal fentanyl alone, these findings provide randomised evidence that a simple change in intrathecal opioid choice can meaningfully reduce postoperative opioid requirements. Because intrathecal opioid administration is technically straightforward and does not require additional training or expensive equipment beyond standard spinal anaesthesia, this change may be achievable without major additional infrastructure or workflow redesign [19]. Although this was a single-centre study, the study site shares features common to many high-volume public-sector maternity services in sub-Saharan Africa, including heavy obstetric workload, constrained monitoring capacity, and the need for simple, reliable, ward-compatible analgesic strategies. In addition, because morphine is a relatively low-cost essential medicine, low-dose intrathecal morphine may offer a favourable cost profile in resource-constrained obstetric services, although formal economic evaluation was beyond the scope of this study. Reduced systemic opioid exposure may also be viewed within a broader stewardship framework, since more appropriate opioid use in acute pain has become an explicit health-system priority [20]. 

This trial was not powered to assess rare adverse events, and it cannot be used to make definitive claims about respiratory safety. At the same time, concern about low-dose neuraxial morphine in healthy obstetric patients should not be detached from obstetric physiology and current practice guidance. Pregnancy is characterised by progesterone-mediated increases in ventilatory drive and enhanced chemosensitivity to carbon dioxide, with a lower maternal PaCO₂ than in the non-pregnant state [21]. These adaptations may partly explain why clinically significant respiratory depression after low-dose neuraxial morphine appears uncommon in healthy obstetric cohorts [5]. SOAP therefore recommends risk-stratified monitoring rather than avoidance of low-dose neuraxial morphine [4]. Our findings should therefore not be interpreted as support for neuraxial morphine in the absence of postoperative observation or escalation capacity. Rather, they support the use of low-dose intrathecal morphine within a service model that can provide basic postoperative monitoring, patient education, dose-limited rescue opioid administration, and clinical escalation when needed. It is not that monitoring is unnecessary, but that fear of rare respiratory events should not, by itself, prevent uptake of low-dose intrathecal morphine where sensible patient selection and observation systems are in place.

### Strengths and limitations

This study has several strengths. It was a randomised comparison of clinically relevant intrathecal regimens conducted in a real public-sector obstetric service rather than in a highly protocolised experimental environment. The primary endpoint, cumulative 24-hour rescue opioid requirement, was pragmatic and clinically meaningful. The consistency of the efficacy signal across the first 12 h, the second 12 h, the full 24-hour period, and the concordant intention-to-treat analysis all support the robustness of the main finding. The pairwise comparisons further strengthened interpretation by showing that the benefit was driven by separation of both morphine groups from fentanyl, rather than by an unstable dose-response effect within the morphine range itself.

The study also has limitations. It was a single-centre study. Although ethics approval and written informed consent preceded enrolment, the trial was registered after participant recruitment had been completed. The trial was participant- and assessor-blinded, but not fully double-blind, because the attending anaesthetist preparing and administering the study drug was aware of treatment allocation. BMI was not available because height was not collected in the original trial dataset. The primary analysis was per protocol because the protocol prespecified exclusion of women in whom valid maternal postoperative follow-up could not be obtained. However, the concordant intention-to-treat analysis indicates that these exclusions did not materially alter the principal efficacy finding. The postoperative regimen reflected local practice at the time of conduct and therefore pre-dated the fully optimised multimodal bundle now recommended by PROSPECT, particularly routine paracetamol and intravenous dexamethasone [3]. This limits direct comparability with contemporary enhanced-recovery pathways, although it also increases the pragmatic relevance of the findings for hospitals in which full multimodal implementation remains incomplete. A more optimised background regimen would likely reduce absolute opioid requirements in all groups and may attenuate the magnitude of the difference between intrathecal morphine and fentanyl. Equally, the clear opioid-sparing signal observed here despite a less complete multimodal background regimen supports the real-world value of low-dose intrathecal morphine in low-resource obstetric settings. In addition, because all women had access to PCA morphine, pain scores, perceived pain relief and desire for additional pain treatment were inevitably influenced by rescue self-titration, which may have compressed between-group differences in patient-reported pain outcomes. Preoperative opioid exposure was not independently verified, and therefore confirmed opioid-naïve status cannot be assumed for every participant. Infants were not systematically assessed for opioid exposure-related outcomes such as excessive sleepiness, limpness, feeding difficulty, or breathing problems, therefore neonatal safety outcomes cannot be inferred from this study. While, PCA use was standardised and reinforced, formal adherence metrics beyond pump-recorded morphine consumption were not collected. Finally, the study was not powered for uncommon adverse events, and safety observations should therefore remain descriptive rather than definitive.

## Conclusion

In women undergoing caesarean delivery in a South African public-sector hospital representative of a resource-constrained obstetric service, intrathecal morphine at both 50 µg and 100 µg reduced 24-hour postoperative IV PCA morphine requirements compared with intrathecal fentanyl 25 µg. The pairwise analyses show that both low-dose morphine regimens separated clearly from fentanyl across all postoperative time periods, while 50 µg and 100 µg did not separate from one another. These findings support 50 µg intrathecal morphine as an effective, pragmatic, and implementable option for post-caesarean analgesia in African and other low-resource settings.

## Supplementary Information

Supplementary Material 1.

Supplementary Material 2.

## Data Availability

The datasets generated and analysed during the current study are not publicly available but are available from the corresponding author on reasonable request.

## Abbreviations

IV: Intravenous
NRS: Numerical Rating Scale
PCA: Patient-controlled Analgesia
