# Supplementary material for: Low-dose intrathecal morphine versus intrathecal fentanyl for post-caesarean analgesia in a resource-constrained setting: a pragmatic randomised trial
Source: BMC Anesthesiol. 2026 Jun 23;26:549. doi: 10.1186/s12871-026-04034-0 (PMC13543457; doi:10.1186/s12871-026-04034-0)
Supplement: Supplementary file 1 [file 12871_2026_4034_MOESM1_ESM.docx]

Supplementary Figures

**Supplementary figures**

**Supplementary Figure S1.** Box plot of IV PCA morphine consumption during 0-12 hours by randomised group

**Supplementary Figure S2.** Box plot of IV PCA morphine consumption during 12-24 hours by randomised group
