# Supplementary material for: Low-dose intrathecal morphine versus intrathecal fentanyl for post-caesarean analgesia in a resource-constrained setting: a pragmatic randomised trial
Source: BMC Anesthesiol. 2026 Jun 23;26:549. doi: 10.1186/s12871-026-04034-0 (PMC13543457; doi:10.1186/s12871-026-04034-0)
Supplement: Supplementary file 2 [file 12871_2026_4034_MOESM2_ESM.docx]

**Supplementary Tables**

**Supplementary Table S1 :** Descriptive safety observations over 24 hours by randomised group

| **Outcome** | **M100 (n = 32)** | **M50 (n = 29)** | **F25 (n = 32)** |
| --- | --- | --- | --- |
| Any nausea/vomiting^#^ | 9/32 (28.1%) | 9/29 (31.0%) | 9/32 (28.1%) |
| Severe nausea/vomiting^#^ | 5/32 (15.6%) | 0/29 (0.0%) | 5/32 (15.6%) |
| Antiemetic treatment | 5/32 (15.6%) | 1/29 (3.4%) | 4/32 (12.5%) |
| Any pruritus^&^ | 12/32 (37.5%) | 14/29 (48.3%) | 9/32 (28.1%) |
| Severe pruritus^&^ | 1/32 (3.1%) | 1/29 (3.4%) | 1/32 (3.1%) |
| Treatment for pruritus | 1/32 (3.1%) | 0/29 (0.0%) | 0/32 (0.0%) |
| Any sedation^!!^ | 7/32 (21.9%) | 7/29 (24.1%) | 10/32 (31.2%) |
| Clinically significant sedation^!!^ | 0/32 (0.0%) | 0/29 (0.0%) | 1/32 (3.1%) |
| Respiratory depression, RR < 8/min^%^ | 0/32 (0.0%) | 0/29 (0.0%) | 0/32 (0.0%) |

Values are n/N (%). Safety outcomes are presented descriptively because the study was powered for analgesic efficacy and not for uncommon adverse events.

\# “Any” nausea/vomiting includes participants with nausea/vomiting scores of 1–3, and severe nausea/vomiting was defined as score 3.

& “Any” pruritus includes participants with pruritus scores of 1–2, and severe pruritus was defined as score 2.

!! “Any” sedation includes participants with sedation scores of 1–3, and clinically significant sedation was defined as score 3, indicating that the participant was difficult to arouse.

% Respiratory depression was defined as respiratory rate < 8 breaths/min. RR, respiratory rate.
